# Use of a Web Portal for Support and Research After a Disaster: Opportunities and Lessons Learned

**DOI:** 10.2196/ijmr.1588

**Published:** 2012-11-21

**Authors:** Geertruid MH Marres, Luke PH Leenen, Johannes W van der Slikke, Eric Vermetten

**Affiliations:** 1 University Medical Centre UtechtCentral Military HospitalMajor Incident HospitalUtrechtNetherlands; 2 VU University Medical CentreAmsterdamNetherlands

**Keywords:** Disaster medicine, Stress Disorders, Post-Traumatic, Internet, mental health, health surveys, stress, psychological, Online Systems, Self-Help groups

## Abstract

**Background:**

In this report we describe the development and use of a web portal in the aftermath of the 2004 tsunami. This large scale disaster confronted many displaced people with death, despair and need for information and support. Awareness and insight in the emotional impact of disasters can provide opportunities for surveillance and early treatment. Moreover, online support systems can contribute to community building, empowerment of victims and resilience.

**Objective:**

We evaluate the development and use of a multilingual web portal that combined a platform for information, emotional support, self assessment and referral with research opportunities. The rapid development, use, advantages, difficulties and learning points are discussed.

**Methods:**

A multidisciplinary working group from the University Medical Centre Utrecht, the Major Incident Hospital and the Central Military Hospital developed a web portal for tsunami victims. The webportal combined: (1) a forum aimed at community building, (2) self assessment tools that in the same time function as a reseach survey, (3) e-consultation, and (4) an information portal.

**Results:**

Within 3 weeks after the tsunami, the working group launched an open, online service (www.TISEI.org. Tsunami Intrenational Survey on Emotional Impact) to foster community) support in the aftermath of the disaster. It combined four functionalities that were earlier previously only used separately. The portal had over 36.800 unique visitors in the first two years. At least 31% (144/464) percent of the Dutch surviving victims could be reached for a survey through the site. The TISEI-environment was available in 15 languages and visitors came from all over the world. Ninety-five percent of all visitors came from Europe or the United States. Subsequent to immediate disaster support, the web portal also served as a memorial archive for anniversary meetings and follow-up incentives. Difficulties we experienced were lack of funding, time pressure, victim-anonymisation, international collaboration and long term maintenance.

**Conclusions:**

A multilingual website with combined modalities for emotional care and research after a natural disaster proved feasible. Web based services like www.TISEI.org in the aftermath of mass disasters can help community building and deliver low level, patient centred and easily accessible information and care. A multilingual website with combined modalities for emotional care and research after a natural disaster proved feasible. Growing Internet penetration world wide and especially the rapid expansion and influence of online communities enables delivery of care and perform research with the internetInternet as a platform. The unpredictable nature of disaster does put time pressure on the development of online solutions and influenced the yield of our site. This highlights the necessity of developing methods and (inter) national collaborations in advance, secure funding, and learn from earlier initiatives.

##  Introduction

On December 26^th^ 2004, a massive undersea earthquake caused a giant shockwave or tsunami that devastated the shorelines of Indonesia, Sri Lanka, India, Thailand and many other countries. More than two hundred thousand people from all over the world were reported missing or dead. Most victims were local citizens but many foreign tourists celebrating Christmas holidays were also hit. Large numbers of survivors were repatriated to their home countries. Among the 500 Dutch visitors in the region, 36 were killed. Several were wounded and were repatriated home in the following days. On January 1^st^ 2005, 23 injured survivors were flown to the Major Incident Hospital (MIH) in Utrecht, the Netherlands. A spontaneous call for a communication platform was heard, in which survivors would hope to connect with the community they were part of as well as other survivors. In the days after the tsunami, numerous web logs and local initiatives were posted. In response to the call for help from survivors, we decided to initiate an online resource providing disaster information, to enable contact among affected persons, and facilitate public connection with mental health professionals.

In disasters, apart from the immediate consequences reflected in the death toll, physical injuries, destruction, and economic consequences, the psychological impact of exposure to a traumatic event can lead to prolonged consequences for mental health. The most notably documented effect of coping with trauma is post-traumatic stress disorder (PTSD) and depression [1,2].

Earlier studies demonstrated that acknowledgement of other survivors can reduce feelings of isolation and have a preventive effect on development of psychological problems [3]. Fellow survivors can provide mutual support, which is often perceived to be more meaningful than help from others who have not experienced the same traumatic events. Online support systems can facilitate this community building by providing a channel for communication amongst victims and health professionals. The value to participants of such virtual forums designed to give and receive emotional support has been documented, although the effect of online communities in “ regular “ mental health care per se has not been equivocally established with high quality scientific evidence yet [4-7].

The psychological aftermath associated with disasters can be managed via a public health approach. Existing services can extend to target mental health care as well as the distribution and availability of resources. The rapid growth of Internet and influence of online communities already in our lives illustrate its opportunities to foster resilience and deliver care after catastrophic events.

Besides mutual support, the Internet can enable self-assessment [8,9] and self-referral. This can stimulate empowerment and a kind of self-triage, which could prevent development of diseases like PTSD and depression in the longer term. Furthermore it could be an instrument to assess the emotional impact of the Tsunami on a group level. Earlier online initiatives in the aftermath of disasters, like surveys after “9/11”[10,11] show the potential of the Internet for health and disaster related research. Awareness and insight in psychological impact of natural disasters on the survivors and the community as a whole has the potential to improve quality of information and support and to aid in planning early treatment options.

In this paper we report the development and use of a web portal as an aid in the management of emotional impact after the Tsunami disaster. We evaluate the feasibility, launching speed, and the development process of a multilingual site that combined a platform for emotional support, self-assessment and referral with research opportunities. The strengths, the faced difficulties and our learning points are discussed.

## Methods

Two days after admission of the patient group to the MIH the chairman of the executive board of the University Medical Centre Utrecht (UMCU) granted support to develop the web portal project. Funding for building and hosting the website was not sufficient at the time we started but was gradually formed along the way. Most of the researchers were voluntary clinicians (psychiatrists, surgeons, psychologists) and IT-specialists. Within 3 weeks after the Tsunami, an open, online service (www.TISEI.org) was launched to foster (community) support in the aftermath of the disaster.

**Figure 1:**
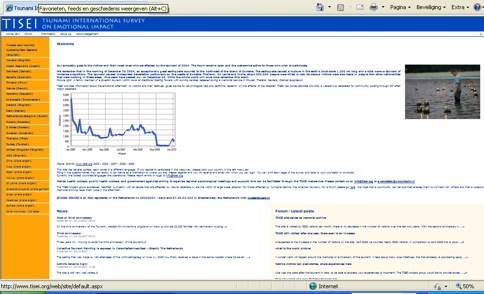
Example of one of the pages of TISEI environment (www.TISEI.org), with multilingual portals (15 different languages), the forum and information portal. The self-assessment/survey and e-consult options are visible in the language portals.

We developed a web service with four functionalities (Figure 1): 1) information portal; 2) forum; 3) self-assessment, and 4) e-consultation. The following describes each of the functionalities in more detail.

### Information Portal

The purpose of the portal was to provide disaster-specific and relevant information. Numerous websites provide information about the impact of disasters. By making a specific disaster based service, the site targets victims of this particular disaster and keeps them up to date. In addition we selected services that provided health related information.

### Forum

The forum facilitates building a community of support for survivors and their loved-ones. This could be through telling personal experiences of the disaster by choosing the web service as an open diary. This general forum allowed for both public and private conversations. Participants had the option to use the ‘back channel’ after registering, which enabled them to communicate with their registered peers only. The forum was intended for victims, support groups, helpers and others to share their concerns and express their feelings. A webmaster monitored content and language in the forums. Each language had its own forum.

### Self-assessment and “Open” Web-based Survey

This functionality facilitates confidential self-assessment and offers a way to understand feelings through a series of scientifically validated questionnaires. 

The survey was tailored to assess the victims’ mental health and to obtain a reasonably reliable recommendation to seek psychological help when necessary. The web survey provided feedback and allowed the participants to print the questionnaire to keep for themselves or take to their counselor or physician. The online forms could also be used as an e-Consult tool on the site. Furthermore results allow insight in mental health across the entire gruop and insight in development of symptoms over time (cross-sectional and longitudinal research). The survey began with questions about demographics, pre-exposure health, and specifics about the stay in South East Asia. These were followed by a compilation of the validated, existing questionnaires and non-validated questionnaires that could reveal valuable information related to mental health problems, such as sleeping problems. Among the structured questionnaires were assessments of the impact of events [12], trauma [13], peritraumatic dissociation [14], emotions, general health, sleep [15] and of depression [16]. For those who had lost loved ones, a grief questionnaire was also provided [17]. After 6 months, a quality of life scale was added [18] but this was soon removed due to copyright issues.

Prior to starting the self-assessment, participants had to give informed consent and register to receive a password by email to enter the survey. Further details of the design of the survey module are depicted in Multimedia Appendix 1 according to the relevant parts of the CHERRIES checklist [19].

### e-Consultation 

This is a functionality for online confidential consultation of professional mental health workers. It offers easily accessible advice on mental health issues, including personal advice about the necessity and location of treatment. It enables victims to seek help in response to symptoms of emotional distress either through online consultation or referral to a counsellor in their own region. The service request would be posted anonymously by email, forwarded to a mobile phone of the on call team, and followed up by email. E-Consult use was only available after registration to the web service and after completion of the web survey. This part could be hosted separately in different portals to accommodate victims with advice relevant to their region of residency.

Server capacity was arranged with external providers. Building and hosting of the actual site was outsourced to a company that builds Internet solutions that have assisted in earlier projects. The site was hosted in the Netherlands and mirrored to warrant data integrity. The framework of the site was language independent, which made it possible to create a local version within a day and make quick multilingual changes.

All personal information was secured with logins verified by registered email and therefore accessible only for the individual participant. Personal information that was entered in the web survey was anonymous through a pseudo anonymisation procedure that meets the criteria of European law. Only the participant and the researchers could retrieve the results of the survey.

The database was accessible for the research team through a password secured login. For the content of the web survey we adapted the questionnaires to Internet research and applied for permission for copyright measures. Professional translators assisted us to develop multilingual portals.

The entire TISEI-environment was language and region independent. Web platforms for online support were made available in 15 languages including: English, French, Spanish, German, Italian, Russian, Dutch, Danish, Finnish, Swedish, Turkish, Czech, Korean, Thai and Indonesian.

Each language portal could have its own user group that could be modified and shaped to a preferred style, with local information and services—as long as the Web surveys remained similar. Each user group could build a database of Web surveys of its own. By building a local team of consultants, each portal could offer local users in different countries the opportunity to provide regional psychological support through the electronic consultation service.

When the setup was finished, the Institutional Review Board of the UMCU assessed and approved the developed website format and study protocol of the survey according to prevailing ethical standards.

Within two weeks the prototype of the site was tested with different Internet providers and computers. We tested ease of use, clarity of instructions, safety, and user friendliness.

Potential site users and participants in the survey were recruited through several means. We attached keywords to relate to search engines. We issued a press release and international news media and organisations such as the International Society for Traumatic Stress Studies (ISTSS), the World Health Organization, World Psychiatric Association and the United Nations were contacted.

## Results

### Visitors to the Website

**Figure 2:**
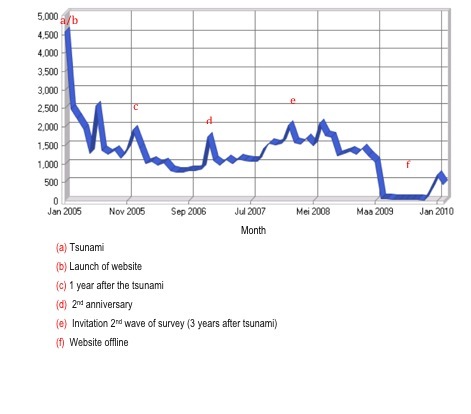
Number of visitors per month on the TISEI site.

**Figure 3:**
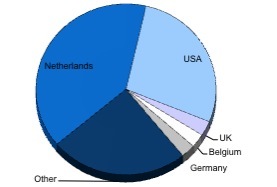
Activity on the site depicted by country of origin.

The total number of unique visitors in the first two years was 36, 849 (January 14, 2005 until January 1, 2007). This was based on unique IP addresses entering the site. These numbers peaked in January 2005 with 4.587 visitors within the first 18 days of the website creation. The average number of visitors per month was 1473 varying between 4587/month and 789/month. The first quarter of 2006 – one year after the Tsunami – showed an upward trend in terms of visitors, hits, and bandwidth compared to the eleven months before. This was even more apparent in December 2007 (Figure 2). The European countries and the United States together made up 95% of all visitors to the site. Most of the European visitors were Dutch (Figure 3).

Links on other websites did not result in substantial visitor recruitment; they contributed to less than 2% of the total number of visitors. Most visitors of the TISEI environment directly went to the URL or found the site when searching the keywords “tsunami” (in different languages including English, Dutch, and Turkish), “TISEI”, or names of victims. 

### Forum

The forum contained many individual stories of victims, as well as responses of people who read the narratives and offered to act as a sounding board or to give explicit help. Examples of these narratives were more extensively described elsewhere [20]. They expressed their symptoms, concerns, complaints, shared evaluation of initiatives they had undertaken for treatment, and provided mutual support to other victims. Experiences and emotions were also expressed by children , illustrated by this contribution on the public part of the site: “I read your story (...) and began to cry. It was so familiar to me! I was also in Thailand during the tsunami, I think it’s so brave of you to talk about it. I would like to talk about it with people our age, I’ve been looking for just that and then I found this Website. It’s good for me to know that I am not the only one with this awful story, I hope we can email (etc) with each other”(www.tisei.org).

It was difficult to come to agreement with international partners to standardize the delivery method for the survey and e-Consult parts of the site. For example Sweden opened a portal but decided to perform a paper and pencil questionnaire instead of participation in the online survey.

For this paper, we only analysed use of the confidential part of the site in our own Dutch portal.

### Self Assessment and Survey: the Dutch Sample

The web-based questionnaires were in a password protected area of the site. Informed consent to include survey results in the database measuring emotional impact was obtained from all participants. 

The number of Dutch citizens in the Tsunami area at the time of the disaster was estimated to a number of 500, of which 36 died in Asia. Through the Dutch portal, 144 people participated in the TISEI survey and filled in 175 assessments. These people were not directly invited by email but encountered the survey on the site independently.

The response rate was expressed as the number of people completing the questionnaire divided by those who viewed it [21], view rate (ratio of the number of unique site visitors / the number of unique survey visitors) [19], or participation rate (ratio of the number of unique survey page visitors / number of people who agreed to participate) [19] could not be calculated in retrospect due to technical limitations of the site. The completion rate expressed as the number of people completing the questionnaire divided by those who agreed to participate [19] it was 95%. 

After three years a secondary measurement was performed in which 39 Dutch survivors participated. However, matching the results of participants over time proved to be difficult due to the method of anonymisation.

### e-Consult

As referral or treatment advice is a local or national issue, this part was supported by each country itself with its own “back office” with their own arrangements with actual care providers. In the Dutch sample 31 people used this modality to consult a professional mental health care worker. All questions could be answered within 24 hrs, except for 3 questions posed in a period of a sick consulent and a non-redirection situation of emails. Besides that, the e-Consult functionality worked well and we did not meet any technical problems with this feature. 

After two months a lot of patients expressed PTSD associated symptoms like insomnia, nightmares, irritability, avoidance and flashbacks. Even after one and two years people contacted through e-consult of the TISEI site looking for help for nightmares, anxiety, concentration problems and weight loss related to the Tsunami.

Twenty one persons were referred through the site to a mental health care worker for treatment or further advice. Other people could be helped through online advice and sometimes repetitive contacts. To our surprise, some of the ‘ referrals’ to care providers were done by pears through the forum. 

###  Satisfaction and Feedback of Visitors

Satisfaction about the website was not quantitatively measured on the website but we received numerous emails of users as well as positive reactions on the forum. The patients admitted to the MIH completed an inquiry and were very positive. The site was rated at 8/10. The information portal was valued as the least part. Improvement ideas from users regarded graphical issues and e.g. a counter of messages and more information on e.g. coping with stress and sleeping problems directly on the site in stead of providing links.

## Discussion

### Opportunities

The TISEI web portal was the first to combine different features of online services used separately in earlier online post disaster initiatives [9]. It fosters community building in conjunction to self assessment questionnaires, e-consultation for referral options, and a research survey. All four different functionalities together can enhance usability and outreach. In this way the portal could serve as a basis for support without interference of health care providers, but with a coupled gateway to professional mental health care and research.

Self-help stimulation through providing adequate, relevant information and a forum serves to empower victims [22,23]. Feelings of self-control can be enhanced though recognition and contact with peers to help to process their own emotions and experiences. Knowing that there are other survivors could reduce feelings of isolation and have a preventive effect on psychological problems [3]. Empowerment after traumatising events is a powerful tool to diminish the severity of mental health complications in the acute phase, which helps to avoid development of chronic complaints. Online communities are growing rapidly and are important for people to exchange experiences and seek advice. The communications on the forum show that the objective of helping victims cope with the impact of a disaster and to help foster resilience was met [20]. 

Online self-assessment further contributes to this by offering people an easily accessible, flexible, and anonymous tool to discuss, organize, and assess feelings and own mental health. Earlier studies report that self-assessment or a research survey can also be an intervention. These methods provide an opportunity for emotional expression, cognitive processing, restructuring of the experience, and active coping [9]. Through the TISEI site, several participants reported the same experience and expressed their appreciation of interest in the emotional aspects of their response. 

Internet applications like the TISEI with a survey module generate research opportunities. Rapid response, tracked standardized answers, cost-effectiveness [24], and global reach are just some examples of its advantages [25]. The identification of individuals at risk for PTSD following a disaster may help organizations prevent both the human and the economic costs of the disease [24]. Several studies showed that sensitive topics, such as psychiatric symptoms are more likely to be reported in self-reported assessments than in interview-based assessments. Web-based data collection has a potential to reduce social bias [26-28]. Studies of responses to disasters have an observational and post event nature. This makes it difficult to identify true causal relationships between exposure and observed outcomes. Reducing selective attrition remains a challenge. As in almost every survey after a disaster, TISEI participants form a volunteer sample of convenience rather than probability sample [29]. Using the Internet to deliver these surveys could diminish such bias, as it also includes people that did not seek professional help in addition to referred patients. Combining research with support groups and self assessment might further improve patient recruitment bias compared to a research site alone [21, 30]. People who are not looking for research or self-assessment may come across the survey while visiting the TISEI site for the forum or other information.

However, these applications also present several logistical as well as scientific challenges. Although surveys like TISEI will not provide causal incidence data, empirical evidence and qualitative research of the impact and adjustment process after disaster exposure will aid clinicians in designing interventions for individuals coping with negative outcome [1]. For an in dept understanding of experiences of particular individuals or groups, qualitative aspects are as important as the quantitative values.

This study shows that at least 29% (144/500) of the Dutch Tsunami victims (31% of 464 survivors) from the Netherlands could be reached for participation in the survey through the TISEI site.

### e-Consult

We believe that offering self assessment and/or a survey should be accompanied by e-Consult or phone service for the participants’ safety. Focussing on the details of these topics might exacerbate an already distressed participant [31] and therefore professional help and/or referral options should be arranged for in advance. Patients can then benefit from the information provided through the self-assessment at the same time.

Online consult modalities can offer rapid client centred emotional care. Many clients may not seek help otherwise because of stigma or lack of information. It is an accessible way to pose health related questions to professionals and can assist to seek help in ones own geographic region.

Moreover, the web service was unique in that it was language and region independent. Because the disaster region was a tourist attraction, survivors came from all over the world. To be accessible to this diverse group of survivors it was necessary to translate the TISEI-site into several languages. This differs from many earlier online initiatives such as 9/11 [9, 11] which were made only in English.

Although the TISEI web service was available in 15 languages, 95% of the visitors were of European or US origin. The finding that relatively few survivors from Thailand, Indonesia and Sri Lanka visited the site may be related to a number of factors. First, major global differences in Internet penetration may play a role. Statistics from Internet World Stats [32] reported Internet penetration in the United States in 2006 was 68%. The European average penetration was lower at 36%. Seventy eight percent of Dutch households had Internet access at that time. The areas hit by the tsunami however, had penetration percentages of 9.9 (Asia) and 2.6 (Africa), respectively. Secondly, destruction of the already limited Internet infrastructure by the tsunami will have further lowered Internet availability in Asia and Africa. Thirdly, focus on survival and rebuilding a home and life in this group of survivors might also have been prioritized over personal mental health, in contrast to tourists that returned to their own non-devastated country after the disaster. Given the current and anticipated growth of the Internet, the development of an online multilingual psycho trauma information system holds great promise for the future.

### Lessons Learned

Apart from the opportunities of the TISEI site, we encountered a lot of challenges, both organizational and scientific, along the way. There are several lessons to be learned in relation to the topics of penetration, cross country collaboration, funding, setup of the survey, and time pressure.

#### Penetration

As discussed above, although the site was multilingual and had global potential, most visitors came from Europe and the United States. Apart from the above mentioned reasons, the disappointing global penetration can also be attributed to an inability to establish firm international collaboration and media attention/participant recruitment.

#### Cross Country Collaborations

Cross country collaborations are mandatory to reach the international population, both for victim recruitment and funding, and for hosting of the portal to accommodate victims with an advice relevant to their region of residency. 

For this study, the Swedish, German and Canadian partners came close to collaborating but were stranded on formal bases. It was not clear which organization had ultimate decision power and authority regarding this issue.

#### Funding

It is no surprise that funding is a crucial but rare prerequisite for innovative acute disaster projects like this. For example in the Netherlands 125 Million EUR was collected on a single promotional night, but this money was dedicated strictly to the rebuilding of the local community. No fundraising was successful for tourist victims or for an international service that had not been proven to be effective yet. Waiting for full and secure funding had interfered with timelines as was also experienced in other projects [9]. Securing quick-response funding, time and people to set up the project was a lesser problem. Long term funding to maintain and manage the website, acquiring data and research on the results proved even more challenging. This interfered with site maintenance, follow up recruitment and result analysis. It was only close to five years later that a grant was awarded to further develop the service for future (Dutch) victims of traumatic events. After three years however, we had difficulties to keep the site online while it could have an important function as a memorial archive, as seen with other sites [33]. These narratives could also be seen as a collective history of the disaster, as these are descriptions of people’s unique trauma experiences, which are publicly accessible.

 We were somewhat surprised by the increase in visitors in the first trimester of 2006 and 2007 around the anniversary date of the tsunami-disaster. Even after two years the site had over 1000 unique visitors a month. Experience from previous disasters have shown that long term help is essential, particularly during and around the anniversary of an event and needs to be extended past the first year [34-36]. On the TISEI site after 2 years people still used e-Consult. Prospective 5 year follow up of tsunami victims admitted to the MIH also showed that onset of late symptoms can appear after several years. As the e-Consult feature had to be discontinued at that time, patients reported experiencing difficulty finding the right help.

#### Survey Setup

Due to technical limitations of the initial survey setup, it is difficult to match respondents over time and research possibilities were limited as a consequence. Secure logins were sent to participants by email when they participated in the survey. These could theoretically be used for long-term follow up, but due to the method of anonymisation we used and changes in email addresses, independent entries over time could not be matched to the same patient automatically. Part of them could be matched manually but this was a time consuming effort with incomplete results. For the same reasons, recontraction of survivors was more complicated than expected. Even though multi-timepoint research was possible, true longitudinal follow up was impossible and results could only be analyzed cross-sectionaly. One question list turned out to be an incomplete version and therefore not validated. Not all instruments we wanted to use could be incorporated due to copyright issues.

Unfortunately, the potential for self-selection bias could not be estimated by measuring the response rate, view rate, or participation rate as this was impossible to calculate in retrospect due to technical limitations of the site. Checklists like CHERRIES [19] can not only help to improve papers reporting Web-based surveys but can also help in the starting phase as a checklist for quality of survey set up, together with reports on similar initiatives [9]. At that time we were not aware of the CHERRIES checklist, but participation rates would have been easy to calculate now if we would have made minor adaptations in the site back then.

#### Time Pressure

Many difficulties we experienced arose from the time pressure to make the service available immediately after the unexpected disaster. Timeliness is important though, both for support and research. We managed to launch the site within 3 weeks after the tsunami to foster community support in the aftermath of the disaster.

There was little time to develop a template for the site, design a survey study, select and adapt questionnaires, and get approval of Institutional Review Board. A lot of issues such as copyrights, securing patient information and data, and referral systems had to be sorted out on short notice.

As time proved to be a limiting factor in optimizing the design of the website, the study, and the funding, we now started a project to develop a template site. Although disasters are unpredictable in timing and nature, many factors involved in providing a site for online community building, e-help and research will be similar and can be sorted out in advance to maximize possible yield.

In conclusion, the TISEI project, set up as a multilingual website with combined modalities for psychological support and treatment as well as research in the aftermath of the tsunami disaster proved feasible. It could be launched quickly and was operational within 3 weeks after the disaster. It fostered community building combined with self assessment questionnaires, offered in conjunction with e-Consultation. Combining this psychological support with research proved feasible. Self-assessment served as an ‘emotional thermometer’, and the outcome was fed back to the research participant. All four different functionalities of the site can enhance each other’s usability and potential reach. In this way the portal could function as a basis for support primarily without interference of health care providers, but with a coupled gateway to professional mental health care and research. Combination of information and self assessment with offering of treatment or help is mandatory for patient safety. Furthermore, it can help to identify people that need treatment in an early stage.

Web-based services in the aftermath of mass disasters can be an aid in community building and deliver low level, easily available and survivor centred information and support. It has potential for support, care and research with rapid response, cost- and means-effectiveness and global reach.

Time proved one of the most important factors in optimizing design and implementation of the survey in our study and in literature. Patient privacy should be a prime. Yet, for longitudinal analysis the anonymisation procedure should allow for returning participants to be identified. Securing funding and available people to manage the site and its contents proved challenging. Long term funding and maintenance has to be taken into account as even after a few years people look for help and the narratives serve as a collective history memorial.

Despite hurdles and lack of penetration to a global outreach, the growing Internet penetration as well as the rapid expansion and influence of online communities should be an incentive to further optimize care and perform research with the Internet as a platform. The unpredictable nature of disaster puts time pressure on the development of online solutions and influenced the yield of our site. Our lessons of the tsunami web service highlight the necessity of developing methods and international collaborations in advance, secure funding, and expand the potential to other survivors of mass psycho trauma.

## Multimedia Appendix 1

TISEI; Web-survey of emotional impact; Checklist for Reporting Results of Internet E-Surveys (CHERRIES).

## Abbreviations

TISEI: acronym for Tsunami International Survey on Emotional Impact which means ‘wisdom’ in Japanese language
PTSD: Post Traumatic Stress Disorder
ISTSS: International Society of Traumatic Stress Studies
UMCU: University Medical Centre Utrecht
MIH: Major Incident Hospital
IRB: Institutional Review Board
CHERRIES: Checklist for Reporting Results of Internet E-Surveys
